# Favourable outcomes of extraskeletal Ewing sarcoma treated on a non-dense chemotherapy backbone with inclusion of radiotherapy for local control

**DOI:** 10.3332/ecancer.2025.2046

**Published:** 2025-11-25

**Authors:** Aditi Dwivedi, Badira Cheriyalinkal Parambil, Venkata Rama Mohan Gollamudi, Maya Prasad, Siddhartha Laskar, Nehal Khanna, Jifmi Jose Manjali, Ajay Puri, Sajid Qureshi, Prakash Nayak, Manish Pruthi, Sneha Shah, Mukta Ramadwar, Poonam Panjwani, Vasundara Patel, Akshay Baheti, Girish Chinnaswamy

**Affiliations:** 1 Division of Paediatric Oncology, Tata Memorial Centre, Homi Bhabha National Institute, Mumbai 400012, India; 2 Department of Radiation Oncology, Tata Memorial Centre, Homi Bhabha National Institute, Mumbai 400012, India; 3 Department of Surgical Oncology, Tata Memorial Centre, Homi Bhabha National Institute, Mumbai 400012, India; 4 Department of Nuclear Medicine, Tata Memorial Centre, Homi Bhabha National Institute, Mumbai 400012, India; 5 Department of Pathology, Tata Memorial Centre, Homi Bhabha National Institute, Mumbai 400012, India; 6 Department of Radiodiagnosis, Tata Memorial Centre, Homi Bhabha National Institute, Mumbai 400012, India

**Keywords:** extra skeletal, Ewing sarcoma, radiotherapy, surgery, outcomes

## Abstract

**Introduction:**

Extra skeletal Ewing sarcoma (EES) has less clearly defined clinical behaviour and treatment strategies. We report the clinical profile, outcomes and prognostic factors of children with EES treated at a single centre over a decade on a non-dose chemotherapy backbone.

**Materials and methods:**

Children ≤15 years of age with EES treated from January 2012 to December 2021 were retrospectively analysed. All received EFT-2001, a non-dose chemotherapy protocol. Local therapy was planned after 9–12 weeks of chemotherapy.

**Results:**

One hundred and six patients formed the study cohort. The majority of the primary was axial in location. Metastasis was present in 25.5%, mainly in the lungs. Local therapy was definitive radiotherapy in 59.1% (*n* = 58), surgery only in 3% (*n* = 3) and surgery and radiotherapy in 37.9% (*n* = 37). At a median follow-up of 43 months (range, 33–52 months), 4-year event-free survival (EFS) and overall survival (OS) of the whole cohort were 70.4% (95% CI: 60.8%–85.3%) and 86.5% (95% CI: 79.3%–94.1%). Four-year EFS and OS of the localised cohort were 73.8% (95% CI: 60%–83.3%) and 88.6% (95% CI: 87.4%–90.2%) and those of the metastatic cohort were 32.4% (95% CI: 21.4%–86.5%) and 61.9% (95% CI: 54.1%–86.3%), respectively. On multivariate analysis, EFS was affected by albumin ≤ 3 g/dL (*p* = 0.01) and delay in any type of local therapy from initiation of chemotherapy ≥12 weeks (*p* = 0.01), whereas OS was affected by male gender (*p* = 0.02).

**Conclusions:**

Survival of children with EES treated on a strategy based on non-dose dense chemotherapy with inclusion of radiotherapy as local control modality is comparable to the Western cohorts, especially in the localised setting. Timely delivery of local therapy is imperative for optimal outcomes.

## Introduction

Ewing sarcoma (ES) is the second most common malignant bone tumour in children and young adults, although, rarely, it may be of extra skeletal origin. Extra skeletal Ewing sarcoma (EES) is a tumour of soft tissues, constituting 20%–30% of ES, with less clearly defined clinical behaviour and treatment strategies [1]. Heterogeneity in chemotherapy protocols can be seen across the globe for this rare entity, varying from an approach similar to skeletal ES to one similar to soft tissue sarcoma with a shorter number of cycles [2, 3]. Likewise, the local therapy approach also varies across co-operative groups and institutions. Although analysis of a large cohort from Surveillance, Epidemiology and End Results Program (SEER) showed comparable outcomes in this subset akin to skeletal ES, there were differences in the early versus late survival between the two cohorts [4]. There were also differences in demographics as well as clinical features, which were noted between these two cohorts in the SEER analysis [4]. The rest of the literature regarding EES is limited to case series and case reports with scarce data from India and the developing world [5]. We report the clinical profile, outcomes and prognostic factors of children with EES treated at a single centre over a decade on a non-dose chemotherapy backbone.

## Materials and methods

### Methods

**Figure 1.**
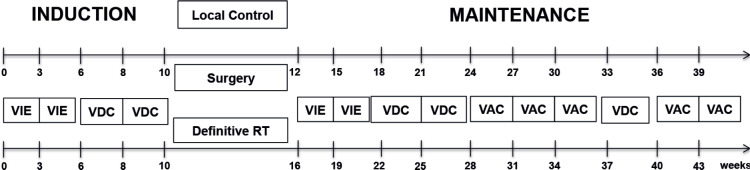
Treatment schema used in the study.

All treatment naïve patients ≤15 years of age with biopsy-proven EES treated at our institute from January 2012 to December 2021 were retrospectively analysed. Diagnosis of EES was established by microscopic and immunohistochemistry evaluation of biopsy samples (NKX2.2, CD99). Molecular analysis using an EWSR1 break‑apart Fluorescence In Situ Hybridisation assay was conducted in a limited number of cases exhibiting diagnostic uncertainty. Also, when poorly differentiated synovial sarcoma was considered in the differential diagnosis, testing for SYT‑SSX1 or SYT‑SSX2 fusion was also performed in a sequential manner. Information on patients was obtained from the electronic medical records and the department database. Patients were staged by Fluoro Deoxy Glucose Positron Emission Contrast Enhanced Computerised Tomography (FDG-PET CECT) as localised or metastatic, with an MRI scan for the primary wherever indicated. Tumour volume was not captured as this was not reported as a routine for the patients, but tumour size was noted. Sites of lung, bone, bone marrow and lymph-node metastasis were determined on PET-CECT as per the affirmative statement of the radiologist. In the metastatic cohort, only lung, lymph-node and oligo-bone metastases (defined as ≤3 sites in earlier cohorts and single bone metastatic sites in later cohorts) were treated with curative intent and included in this study. Single site involvement was defined as isolated lung (regardless of the number of lesions), lymph node, liver or bone involvement. All received EFT-2001 chemotherapy protocol [6]. EFT-2001 protocol consisted of a combination of drug couplets alternately administered on a 3-weekly schedule, except for an interval compression in induction. Details in Figure 1. Granulocyte-Colony Stimulating Factor was not used as primary prophylaxis in this regimen and was used only during periods of febrile neutropenia. The total duration of the regimen was 42 weeks if local therapy was surgery and 46 weeks if definitive radiotherapy (RT) was given. Local therapy (Definitive radiotherapy where R0 resection is not possible or surgery and radiotherapy) was planned after 9–12 weeks of chemotherapy by a multidisciplinary team, with metastatic sites addressed with radiotherapy [6]. Radiotherapy was delivered at a dose of 55.8 Gy in 31# at 1.8 Gy/#, 5 days a week for local control if it was the only modality used (definitive RT) and at 45 Gy for post operative RT at 1.8 Gy/#. All those with lung metastases received lung bath to a dose of 12.6 Gy/7# irrespective of the response and were delivered at the time of definitive RT or following surgery to the primary. If gross residual lung metastasis were present post induction and was surgically resectable, metastatectomy was contemplated prior to lung bath. Bone metastasis was addressed with local RT at a dose of 55.8 Gy and lymph node metastasis received RT to the lymph node region at a dose of 45 Gy. Relapse/Progression was diagnosed as an increase in size as per response evaluation criteria in solid tumors criteria or new lesions as detected by FDG-PET CECT scan and the mass was biopsied only where the diagnosis of disease was doubtful. Primary objectives were to assess event-free survival (EFS) and overall survival (OS). Secondary objectives were to define the demographic and clinical profile as well as to identify prognostic factors.

### Statistical methods

Baseline variables and outcomes were analysed by descriptive statistics. For survival analysis, an event was defined as progression, relapse, development of second malignancy or death due to any cause. EFS was calculated from the date of diagnosis to the event or last follow-up.

OS was calculated from the date of diagnosis to death due to any cause or last follow-up. Estimates of survival were computed using the Kaplan–Meier method. The hazard ratios (HRs) and significance associated with patient characteristics were assessed in a Cox proportional hazards regression model. In multivariate analysis, we included variables with a *p* value < 0.1 on univariate analysis. Statistical analysis was performed using STATA software, version 16.1.

## Results

### Epidemiological and clinical profile

**Figure 2.**
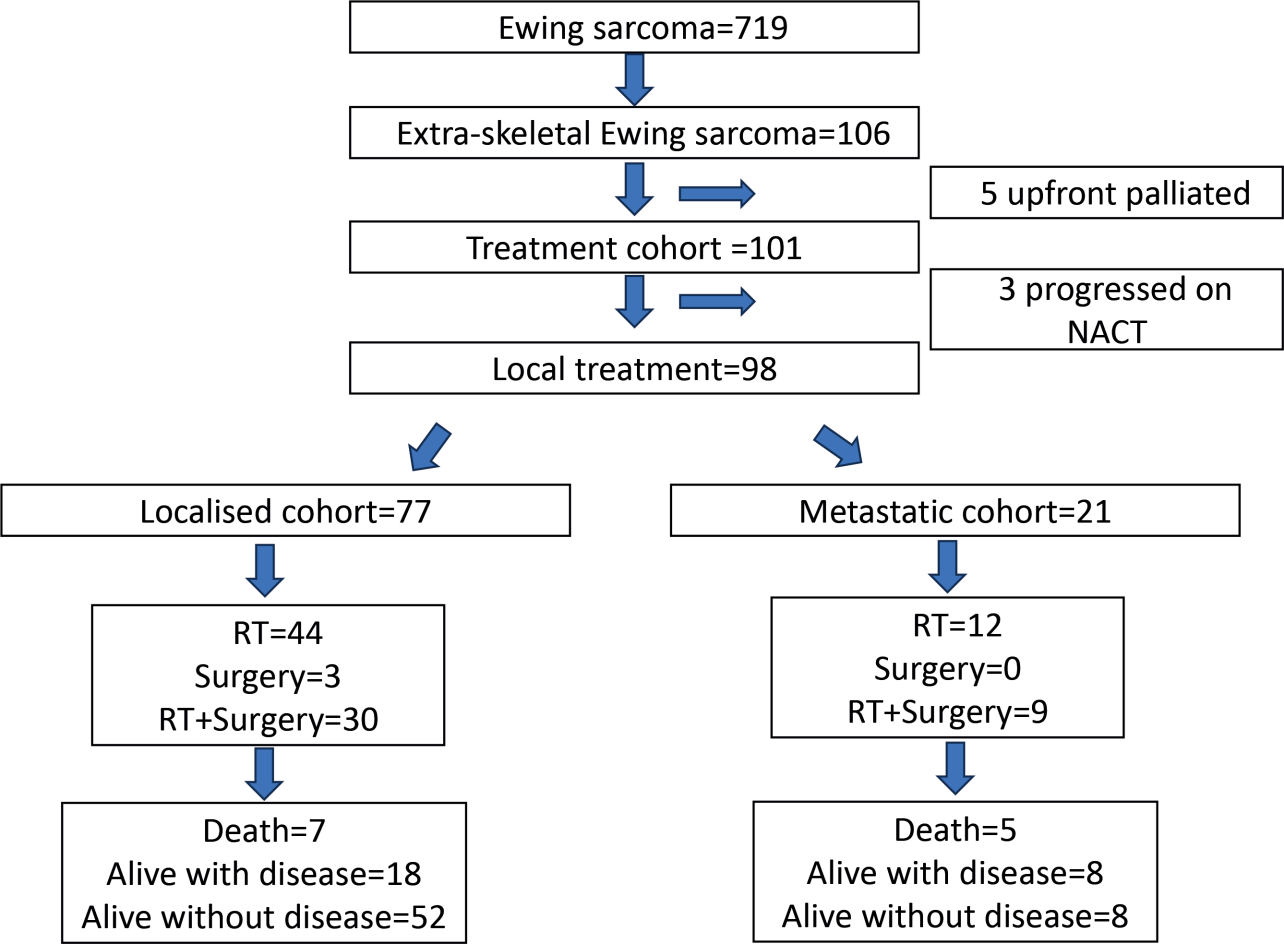
Consort diagram of the study.

One hundred and six patients formed the study cohort, which constituted 14.7% of the treatment naïve ES diagnosed during the period (*n* = 719). The median age was 10 years (range, 0.7–15 years) and male-to-female ratio was 1.2:1. The cohort had a similar distribution of children aged <10 and ≥10 years (50.0% each). The majority of the primary was axial in location (74.5%). Median tumour size (*t* size) was 7.1 cm (range, 2–27 cm). Metastasis was present in 25.5%, mainly in the lungs. Details in Tables 1 and 2. Consort in Figure 2.

### Treatment

In the treated cohort of 101 children (Of total, 106), five were upfront palliated due to disseminated disease and were not included for survival outcome analysis. Three patients progressed on neoadjuvant chemotherapy and were offered palliation. No patient had abandoned while on therapy in this cohort. There were no major treatment interruptions or delays in this cohort. Local treatment to the primary was delivered in 98 patients. This was definitive radiotherapy in 59.1% (*n* = 58), surgery only in 3% (*n* = 3) and surgery and radiotherapy in 37.9% (*n* = 37). Eleven (11.2%) of 98 patients received brachytherapy. In the localised cohort (*n* = 77), the above distribution for type of local therapy was definitive radiotherapy in 57.1% (*n* = 44), surgery only in 3.9% (*n* = 3) and surgery and radiotherapy in 39.0% (*n* = 30), respectively, and for the metastatic cohort (*n* = 21) this was definitive radiotherapy in 57.1% (*n* = 12), surgery only in none and surgery and radiotherapy in 42.9% (*n* = 9), respectively.

Of the 42 patients who underwent surgery post-induction chemotherapy, histopathological response was available for 31 patients. Necrosis was 100% in 16.1% (*n* = 5), 90%–99.9% in 29% (*n* = 9) and <90% in 54.8% (*n* = 17) patients.

### Outcomes

At the time of analysis, 86 were alive (there were 30 patients with a follow-up date more than a year at the time of analysis) and 15 expired. There were 35 disease-related events in the treated cohort, which were progression while on chemo in 25.7% (*n* = 9), early relapse in 34.3% (*n* = 12) (defined as Disease Free Interval (DFI) < 2 years) and late relapse in 40% (*n* = 14) (defined as DFI ≥ 2 years). Details in Table 3. and Non-relapse mortality in the whole cohort was 2.9% (*n* = 3) (cardiomyopathy-66.6%, *n* = 2: sepsis-33.3%, *n* = 1).

**Figure 3.**
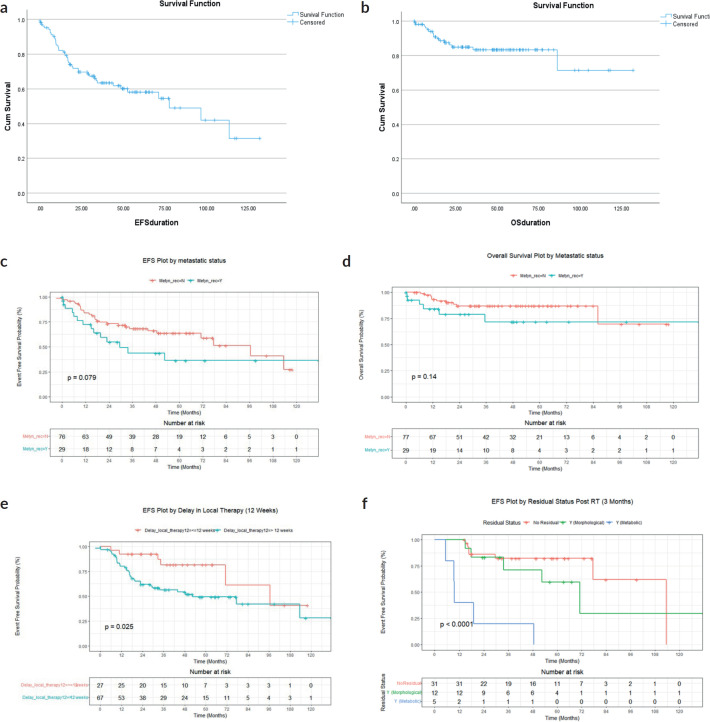
(a) EFS of whole cohort (b) OS of whole cohort (c) EFS of localised and metastatic cohort (d) OS of localised and metastatic cohort (e) EFS based on delay in local therapy for the whole cohort (f) EFS based on post definitive RT FDG-PET residual status for the whole cohort.

At a median follow-up of 43 months (range, 33–52 months), 4-year EFS and OS of the whole cohort were 70.4% (95% CI: 60.8%–85.3%) and 86.5% (95% CI: 79.3%–94.1%). Four-year EFS and OS of the localised cohort were 73.8% (95% CI: 60%–83.3%) and 88.6% (95% CI: 87.4%–90.2%) and of the metastatic cohort were 32.4% (95% CI: 21.4%–86.5%) and 61.9% (95% CI: 54.1%–86.3%), respectively. Survival curves in Figure 3.

In the metastatic cohort, 4-year EFS and OS of those with isolated pulmonary metastases were 33.6% (95% CI: 21%–82%) and 65.6% (95% CI: 43%–100%), respectively, while 4-year EFS and OS of those with extrapulmonary metastases were 18.8% (95% CI: 12%–88%) and 39.8% (95% CI: 26%–100%), respectively.

Of the 48 patients who had 3 months post-definitive radiotherapy PET-CT scan (FDG-PET CT scan was done in all patients who received definitive RT to assess for residual lesion as a uniform departmental policy 3 months after completion of definitive RT), no residual was documented in 64.5% (*n* = 31), while morphological only residual without FDG activity was present in 25% (*n* = 12) and FDG-avid residual in 10.4% (*n* = 5). Four-year EFS for the above three groups were 82.4% (95% CI: 69.4%–97.7%), 71.4% (95% CI: 48.2%–100%) and 20% (95% CI: 3.4%–100%), *p* < 0.001, respectively. Four-year OS were 95.7% (95% CI: 87.6%–100%), 91.7% (95% CI: 77.2%–100%) and 60% (29.3%, −100%), *p* = 0.003, respectively.

### Prognostic factors

For the whole cohort, on univariate analysis, male gender (44.3% versus 57.7%, *p* = 0.045), albumin ≤ 3 g/dL (39% versus 61% *p* = 0.007), delay in any type of local therapy from initiation of chemotherapy >12 weeks (18.3% versus 81.7%, *p* = 0.03) and presence of any residual disease (morphologic or metabolic) on PET-CT scan 3 months post definitive radiotherapy (31% versus 69%, *p* = 0.01) adversely affected EFS whereas male gender (44.3% versus 57.7%, *p* = 0.045), delay in any type of local therapy from initiation of chemotherapy ≥12 weeks (18.5% versus 81.5%, *p* = 0.04), presence of lung metastases (*p* = 0.04) adversely affected OS. On multivariate analysis, EFS was affected by albumin ≤ 3 g/dL (*p* = 0.01) and delay in any type of local therapy from initiation of chemotherapy ≥12 weeks (*p* = 0.01), whereas OS was affected by male gender (*p* = 0.02). Details in Tables 4–7.

For the localised cohort, EFS was affected by the location of tumour (axial versus nonaxial) (37.7% versus 62.3%, *p* = 0.04), albumin ≤ 3 g/dL (42% versus 58%, *p* = 0.02), delay in any type of local therapy from initiation of chemotherapy ≥ 12 weeks (15.7% versus 84.3%, *p* = 0.01) on univariate analysis. Overall Survival in this cohort was affected by duration of symptoms (15% versus 85%, *p* = 0.03) on univariate analysis. Multivariate analysis was not possible in localised and metastatic cohorts, as there were not enough events in each of these groups when independently analysed.

For the metastatic cohort, EFS was affected by age >10 years (38.9% versus 61.1%, *p* = 0.01), male gender (41.4% versus 58.6%, *p* = 0.03), duration of symptoms in months (27.4% versus 72.6%, *p* = 0.02), tumour size ≥ 8 cm (26% versus 74%, *p* = 0.02) on univariate analysis. OS was affected by age > 10 years (36.4% versus 63.6%, *p* = 0.01), duration of symptoms (27.3% versus 72.7%, *p* = 0.03) on univariate analysis.

## Discussion

The overall proportion of extraosseous origin of ES was relatively less (14.7%) compared to western cohorts (20%–31%), though akin to another EES cohort described from India (16%) [2, 4–7]. The clinico-epidemiological profile of our EES cohort had similarities with the SEER EES cohort, though SEER cohort included patients up to 19 years of age. The prevalence of pelvic primaries (*p* = 0.021) and advanced tumour size, defined as ≥ 10 cm (*p* = 0.025), was less in our study compared to the SEER cohort [4]. The reason for the lesser proportion of tumours ≥ 10 cm compared to the western cohorts is unclear [4, 6]. The location of the primaries in thorax, pelvis and paravertebral regions eliciting early compressive and obstructive presentations in two-third of the patients may be a contributing factor for this. Nevertheless, children with *t*-size > 8 cm was similar in proportion to the published earlier EES cohorts [2, 6, 7]. Our EES cohort also had more axial primaries compared to the skeletal ES cohort (*p* < 0.001) treated at our institute, commensurate with published literature [6].

The survival outcomes at a median follow-up of 43 months are relatively fair compared to the 5-year EFS and OS published in literature for EES and skeletal ES (including the cohort previously reported from our institute on a non-dose dense chemotherapy backbone), though long-term follow-up is warranted [2, 6, 7]. Notably, EFS and OS for all the subsets in our study are superior to that reported from another EES cohort from India, with a relatively similar clinical profile treated on a 3-weekly alternating Vincristine Adriamycin Cyclophosphamide/ Ifosphamide Etoposide chemotherapy [5]. These outcomes on our study are favourable taking cognizant of the fact that, nearly half of the cases had tumour size >8 cm and the proportion of poor responders with tumour necrosis <90% on post neoadjuvant surgical specimens is significantly higher compared to the skeletal ES cohort treated on the same treatment protocol (*p* = 0.007) [6]. There is no literature available on tumour necrosis for EES cohorts, though radiological response rates have been enumerated as being higher than the skeletal ES cohorts [5, 7]. Also, surgical resection of the primary was undertaken in only one-third of our children compared to nearly three-fourths of paediatric cases of EES in western cohorts, probably reflecting the site of primary precluding the possibility of R0 resection [2, 4]. Radiotherapy was administered in nearly all our patients (97%) vis a vis up to 50% in published cohorts [2]. These evident differences in the modality of local control, with application of RT in all, might be the reason for the comparable outcomes despite an increased proportion of poor responders in the evaluable population. Though the timing of delivery of RT post-surgery did not impact outcomes, a delay >12 weeks in the delivery of any type of local therapy from the initiation of chemotherapy adversely affected outcomes in both the whole and localised cohorts. This is a logistical and real concern when the resources are limited and needs addressal to ensure desirable outcomes. The outcomes of metastatic cohorts were dismal and similar to the documented EFS and OS in literature, with single site, especially lung only metastasis faring better and can be triaged for treatment and fund allocation in resource-constrained settings in Low-Middle Income Countries (LMICs) [2]. The differential outcomes based on extraosseous origin of the western cohorts treated on interval compressed chemotherapy is not available for comparison.

Predictably, those with *t*-size > 8 cm had worse EFS in the whole and localised cohorts, though OS was not affected significantly. Also, males had worse outcomes in conformity with earlier studies [4]. In addition to the factors described above, baseline low serum albumin resulted in unfavourable EFS across the cohorts. This has been previously also stated in skeletal ES and EES studies published from India, but whether this is reflective of malnutrition prevalent in the community or part of the inflammatory state associated with ES is unclear [8]. Another factor determinant of poor outcomes in the whole and localised cohorts was the presence of any type of residual (morphologic only or FDG avid residual) on post RT 3-month FDG PET scan in those who received definitive RT as local therapy. Though the number of patients evaluable for the same was limited, there was a striking difference in outcomes, especially in those with FDG-avid residual.

The study has its own limitations. This is a retrospective study of a small sample size, limited by the non-availability of toxicity data. Molecular testing was done only in selected cases with diagnostic confusion. Also, long-term outcomes of this cohort need follow-up. Despite these, this cohort represents a single centre experience of a rare cohort treated on a uniform non-dose dense chemotherapy protocol. Outcomes are relatively fair compared to the dose-dense approach, facilitating the practical application of this treatment strategy in LMICs where supportive care facilities, increased background multi-drug-resistant infections, undernutrition and toxicities are a concern, restricting the application of intensive chemotherapy. In addition, this study also analyses tumour necrosis data, which is hitherto not available in EES.

## Conclusion

Survival of children with EES treated on a strategy based on non-dose dense chemotherapy with inclusion of RT as local control modality is relatively fair compared to the western cohorts, especially in the localised setting. Timely delivery of local therapy is imperative for optimal outcomes.

## List of abbreviations

EES, extraskeletal Ewing sarcoma; EFS, event-free survival; OS, overall survival; PET-CT, positron emission tomography-computed tomography; PORT, post-operative radiotherapy; RFS, relapse-free survival; RT, radiotherapy.

## Conflicts of interest

The authors declare no conflicts of interest.

## Funding

Nil.

## Data availability statement

The data that support the findings of this study are available from the corresponding author upon reasonable request.

## Figures and Tables

**Table 1.** Patient demographics and clinical characteristics.

Demographic or clinical characteristic	Number of patients (%)
Age at diagnosis, years	
≤5	24 (22.6)
6–9	28 (26.4)
≥10	54 (50.9)
Sex	
Male	59 (55.7)
Female	47 (44.3)
Primary site	
Axial	79 (74.5)
Appendicular	27 (25.4)
Axilla	2 (2.1)
Lung, mediastinum	7 (7.4)
Paravertebral	20 (21.2)
Pelvis	10 (10.6)
Hand	1 (1)
Arm	2 (2.1)
Forearm	2 (2.1)
Parapharynx/larynx	6 (6.3)
Thyroid	1 (1)
Uterus/ovary	2 (2.1)
Neck	2 (2.1)
Thigh	8 (8.4)
Leg	2 (2.1)
Retroperitoneum	3 (3.1)
Chest wall	30 (31.8)
Kidney	6 (6.3)
Prostrate	1 (1)
Orbit	1 (1)
Metastases	
Yes	27 (25.5)
No	79 (74.5)
Site of metastases (out of 27)	
Lung only	13 (48.1)
Bone only	5 (18.5)
Lymph node only (regional)	4 (14.8)
Combined	5 (18.5)
Tumor size	
≥8cm	48 (45.3)
<8cm	58 (54.7)
Median duration of symptoms in months (range)	29 (1–43)
Delay in any local control > 12 weeks	
For the entire cohort (out of evaluable 98 )	32 (31.3)
For the localised cohort (out of evaluable 76)	12 (9.1)

**Table 2.** Comparison of the available clinical characteristics of our study cohort with SEER-EES cohort and the skeletal ES cohort treated at our institute .

Demographic or clinical characteristic	EES-current cohort *N* (%) (2021)	EES-SEER cohort *N* (%) (1973–2007)	*p* value	Skeletal ES- ≤15 years cohort treated at our institute, *N* (%) (2013)	*p* value
Total number	106	683		192	
Sex					
Male	59 (55.7)	365 (53.4)		109 (56.8)	
Female	47 (44.3)	318 (46.6)	0.67	83 (43.2)	0.853
Age					
Median in years (range)	10 (0.7–15)	19.5 (0–39)		11 (0.8–15)	
<10 years	53 (50.0)	-		77 (40.1)	
≥10 years	53 (50.0)	-		115 (59.9)	0.147
Primary site					
Axial	78 (73.9)	498 (72.9)		86 (44.8)	
Appendicular	28 (26.4)	185 (27.1)	0.885	106 (55.2)	<0.001
Pelvic primary					
Pelvic	11 (10.2)	135 (19.8)		33 (17.2)	
Non-pelvic	95 (89.8)	548 (80.2)	0.021	159 (82.8)	0.113
Metastases					
Yes	27 (25.5)	206 (30.1)		40 (20.8)	
No	79 (74.5)	477 (69.9)	0.325	152 (79.2)	0.359
Tumor size					
≥10 cm	31 (28.9)	278 (40.7)	0.025	86 (44.8)	0.009
≥8 cm	48 (45.3)	-		124 (64.6)	0.001
Local therapy	*N* = 98			*N* = 189	
Definitive RT	58 (59.1)	50.1		56 (29.6)	
Surgery + RT	37 (37.9)			67 (35.4)	
Surgery only	3 (3.0)	-		66 (34.9)	0.007
Histological necrosis	*N* = 31			*N* = 121	
100%	5 (16.1)	-		51 (42.1)	
90%–99.9%	9 (29)			36 (29.8)	
<90%	17 (54.8)	-		34 (28.1)	0.007

**Table 3.** Details of relapse in the cohorts.

Variable	Whole cohort *n* = 35	Localised cohort *n* = 26	Metastatic cohort *n* = 9
Timing of relapse			
Progression on chemotherapy	9 (25.7%)	7 (26.9%)	2 (22.2%)
Early relapse	12 (34.2%)	8 (30.7%)	4 (44.4%)
Late relapse	14 (40%)	11 (42.3%)	3 (33.3%)
Type of relapse			
Local	16 (45.7%)	12 (46.1%)	4 (44.4%)
Metastatic	10 (28.5%)	9 (34.6%)	2 (22.2%)
Combined	9 (25.7%)	5 (19.2%)	3 (33.3%)

**Table 4.** Prognostic factors (A) EFS- univariate.

A)									
Variable	HR	95% CI	*p* value	HR	95% CI	*p* value	HR	95% CI	*p* value
	For whole cohort			For localised cohort			For metastatic cohort		
Gender	1.92	1.01−3.66	0.04	1.38	0.64−2.94	0.40	4.09	1.11−14.9	0.03
Axial site	1.44	0.28−1.45	0.37	1.76	0.10−1.11	0.04	0.59	0.71−7.89	0.11
Presence of metastasis	1.67	0.92–3.33	0.12	-	-	-	-	-	-
Age (10 < versus ≥ 10 years)	0.64	0.27−1.53	0.32	0.74	0.26−2.13	0.58	0.09	0.01−0.65	0.01
Local control delay > 12 weeks	2.60	1.09−6.22	0.03	3.75	1.26−10.64	0.01	0.67	0.14−3.18	0.62
Tumor size (<8 versus ≥ 8 cm)	0.73	0.39−1.34	0.31	0.76	0.35−1.66	0.05	0.28	0.09−0.83	0.02
Post definitive RT PET-any residual	1.76	0.55−5.64	0.33	2.66	0.44−15.9	0.28	1.36	0.22−8.22	0.73
Site of metastasis	1.00	0.34−2.92	0.99	-	-	-	0.39	0.05−3.07	0.37
Albumin (>3 versus ≤ 3 g/dL)	0.24	0.08−0.68	0.007	0.25	0.07−0.85	0.02	0.1	0.11−15.6	1.1
Duration of symptoms	1.04	0.95−1.14	0.31	0.85	0.70−1.04	0.11	1.11	1.01−1.22	0.02

**Table 5.** Prognostic factors: EFS- univariate.

Variable	HR	95% CI	*p* value	HR	95% CI	*p* value	HR	95% CI	*p* value
	For whole cohort			For localised cohort			For metastatic cohort		
Gender	3.81	1.08−13.39	0.03	3.29	0.69−15.5	0.13	4.55	0.52−39.83	0.17
Axial site	1.3	0.09−1.89	0.38	1.2	0.19−12.4	0.99	0.46	0.40−13.62	0.13
Presence of metastasis	1.91	0.76−5.82	0.24	-	-	-	-	-	-
Age (10 < versus ≥ 10 years)	0.58	0.15−2.25	0.43	1.70	0.19−14.6	0.62	0.03	0.02−0.472	0.01
Local control delay > 12 weeks	6.03	0.78−46.2	0.08	0.94	1.60−3.40	0.99	0.46	0.04−4.54	0.51
Tumor size (<8 versus ≥ 8 cm)	1.07	0.40−2.86	0.88	1.07	0.30−3.80	0.91	0.50	0.08−2.87	0.43
Post definitive RT PET-any residual	1.13	0.10−12.5	0.91	20.1	1.70−23.7	0.01	13.6	0.12−14.6	1.2
Site of metastasis	0.45	0.08−2.50	0.36	-	-	-	0.94	0.10–8.17	0.95
Albumin (>3 versus ≤ 3 g/dL)	0.28	0.06−1.25	0.09	0.44	0.05−3.59	0.45	0.8	0.2−7.34	1.6
Duration of symptoms	1.09	0.99−1.29	0.07	0.47	0.24−0.94	0.03	1.12	1.00−1.26	0.03

**Table 6.** Prognostic factors: multivariate analysis for EFS-whole cohort.

Variable	HR	95% CI	*p* value
Gender	1.88	0.96–3.69	0.065
Albumin ≤ 3 g/dL	0.37	0.17–0.79	0.011
Local control delay > 12 weeks	3.21	1.31–7.86	0.011

**Table 7.** Prognostic factors: multivariate analysis for OS-whole cohort.

Variable	HR	95% CI	*p* value
Gender	5.90	1.30–2.6	0.02
Local control delay > 12 weeks	7.05	0.94–2.5	0.062
